# Prognostic values and immune suppression of the S100A family in pancreatic cancer

**DOI:** 10.1111/jcmm.16343

**Published:** 2021-02-12

**Authors:** Hongkai Zhuang, Xinming Chen, Fengying Dong, Zedan Zhang, Zixuan Zhou, Zuyi Ma, Shanzhou Huang, Bo Chen, Chuanzhao Zhang, Baohua Hou

**Affiliations:** ^1^ Department of General Surgery Guangdong Provincial People's Hospital Guangdong Academy of Medical Sciences Guangzhou China; ^2^ Shantou University of Medical College Shantou China; ^3^ Department of Hepatobiliary Surgery Shenshan Central Hospital Sun Yat‐Sen Memorial Hospital Sun Yat‐Sen University Shanwei China; ^4^ Forth Department of Geriatrics General Hospital of Southern Theater Command, Pla Guangzhou China; ^5^ Department of Breast Cancer Cancer Center Guangdong Provincial People's Hospital Guangdong Academy of Medical Sciences Guangzhou China

**Keywords:** CD8^+^ T cells, focal adhesion, pancreatic cancer, Ras‐stimulating signalling pathway, S100A family

## Abstract

S100 calcium‐binding protein A (S100A) family members regulate multiple biological functions related to pancreatic cancer (PC) progression and metastasis. However, the prognostic and oncologic values of S100A family have not been systematically investigated in PC. In the present study, the mRNA expression and potential functions of S100A family were investigated by bioinformatic analysis. Our results demonstrated that overexpression of S100A2, S100A6, S100A10, S100A11, S100A14 and S100A16 was significantly associated with higher T stage, advanced histologic grade and worse prognosis in PC. Besides, one CpG of S100A2, three CpG of S100A6, four CpG of S100A10, four CpG of S100A11, two CpG of S100A14 and five CpG of S100A16 were negatively associated with corresponding S100A family members expression and positively associated with overall survival (OS). The signature based on four CpGs showed good prediction ability of OS. Besides, S100A2 overexpression took part in the regulation of mitotic cell cycle, ECM‐receptor interaction and HIF‐1α transcription factor network. Overexpression of S100A6, S100A10, S100A11, S100A14 and S100A16 may impair the infiltration and cytolytic activity of CD8^+^ T cells through focal adhesion‐Ras‐stimulating signalling pathway in PC. Overall, this study explores the multiple prognostic values and oncologic functions of the S100A family in PC.

## 1 INTRODUCTION

As one of the most aggressive tumours, pancreatic cancer (PC) is currently the fourth most common cause of cancer mortality, causing about 4.5% of all cancer induced deaths worldwide.^1^ The overall 5‐year survival rate of PC is less than 8%.^2^ Immune therapy has been a landmark improvement over the last decade for the treatment of various cancers, such as melanoma, non–small‐cell lung cancer and hepatocellular carcinoma.^3^, ^4^, ^5^, ^6^, ^7^, ^8^ However, these immune checkpoint inhibitors have limited effectivities for PC as single agents, due to the limited infiltration and impaired anti‐tumour effect of CD8^+^ T cells in the TME of PC.^9^, ^10^, ^11^ Consequently, the identification of reliable prognostic biomarkers and drug targets involved in the tumorigenesis and immunosuppression of PC is crucial for developing more useful immunotherapeutic drugs for PC patients.

S100 calcium‐binding protein A (S100A) family consists of 17 members (eg S100A1, S100A2, S100A3, S100A4, S100A5, S100A6, S100A7, S100A7A, S100A7L, S100A8, S100A9, S100A10, S100A11, S100A12, S100A13, S100A14 and S100A16), located on chromosomes 1.^12^ S100A family members are reported to regulate multiple biological functions related to PC progression.^13^, ^14^ Previous studies reported that S100A2 and S100A10 are negative prognostic biomarkers in PC.^15^, ^16^ S100A4 was suggested to decrease the BNIP2 expression and contribute to chemoresistance and survival in PC cells.^17^ Deletion of S100A6 is reported to decrease the invasion of PC cells.^18^ S100A11 has been reported to be an unfavourable prognostic factor in PC and promote PC cell proliferation through PI3K‐Akt signalling pathway.^19^ Collectively, the prognostic values and potential function of some of S100A family have been noticed in PC. However, the prognostic and oncologic characterizations of the whole S100A family remains poorly investigated in PC. To note, there is little study about the association between the mRNA expression of S100A family and immune infiltration in PC.

In the present study, using public resources and multiple bioinformatic analysis, we comprehensively assessed the mRNA expression and DNA methylation of S100A family and their prognostic values in PC. We also investigated the potential biological roles of S100A family members in PC using functional enrichment analysis in ConsensuspathDB (http://cpdb.molgen.mpg.de/).^20^ Besides, for the first time, we developed a prognostic signature based on the CpG methylation of S100A family members for OS of patients with PC. Furthermore, with the help of ingle sample Gene Set Enrichment Analysis (ssGSEA), we are also the first to evaluate the potential relationships between S100A family expression and immune cell infiltration levels in PC.

## 2 MATERIALS AND MATHODS

### 2.1 Data acquisition

First, we downloaded the mRNA expression data recorded based on FPKM and corresponding clinical information for PC from the Cancer Genome Atlas (TCGA, https://cancergenome.nih.gov/) database. The CpG methylation data of S100A family members in TCGA PC cohort were downloaded through MethSurv (https://biit.cs.ut.ee/methsurv/). Among the 177 PC cases in TCGA PC cohort, 171 were cases with OS >1 month. Besides, GSE28735 data set were used for paired differential expression analysis for S100A family members. TCGA PC data set and GSE28735 data set are both freely available as public resources. Therefore, local ethics approval was not needed.

### 2.2 Differential expression analysis of S100A family members

First, differential expression analysis of S100A family in PC was performed using the GEPIA database (http://gepia.cancerpku.cn/index.html) based on the TCGA and GTEx projects. Then, the differential expression of S100A family members between PC samples (n = 45) and the corresponding adjacent non‐tumour tissues (n = 45) was investigated using GSE28735 data set. In addition, the overlapped significantly differential expression S100A family members between GEPIA database and GSE28735 database were selected for further analysis.

### 2.3 Survival analysis in Kaplan‐Meier plotter database

The prognostic values of differentially expressed S100A family in PC were analysed using Kaplan‐Meier (KM) plotter database (http://kmplot.com/analysis), a website database based on resources from TCGA database. The final prognostic KM plots were presented with a hazard ratio (HR), 95% confidence interval (CI) and log‐rank P value. A *P* value < .05 was considered statistically significant.

### 2.4 S100A family members in TNM stage and histologic grade

To determine whether S100A family members could promote PC progression, we investigated the expression level of S100A family members in different TNM stage and histologic grade. The differences between the two groups were compared using the Wilcoxon test. And the differences among three or more groups were compared using the Kruskal‐wallis test.

### 2.5 DNA methylation data of S100A family members in MethSurv

CpG methylation data of S100A family members were extracted from MethSurv. Correlation analysis between the mRNA expression of S100A family members and the corresponding CpG methylation in PC was conducted with the threshold of Pearson correlation coefficients <−0.4 and *P* value <.05. Furthermore, we also evaluated prognosis‐associated CpG of S100A family members using univariate Cox analysis and intersected them with the CpG which significantly correlated with the corresponding S100A family member expression. The overlapped CpGs were considered as critical CpGs. KM survival Curves of critical CpGs were conducted using GraphPad Prism 8.0.1 (GraphPad Software, lnc.); the best cut‐off points were determined by the X‐tile 3.6.1 (Yale University, New Haven, CT, USA).^21^

In addition, we performed Lasso regression and multivariate Cox analysis to develop a prognostic signature based on critical CpG. Risk score = ∑ the multivariable Cox regression coefficients × the methylation of critical CpG. High/low‐risk groups were determined according to the best cut‐off point determined by the X‐tile 3.6.1. The predictive ability of the CpG signature was assessed by C‐index, and the AUC of ROC curve. Moreover, gene set enrichment analysis (GSEA) (v3.0, http://software.broadinstitude.org/gsea/) was performed to investigate the differences in potential biological functions between the high‐ and low‐risk groups.

### 2.6 Pathway enrichment analysis of S100A family members

First, co‐expression genes of S100A family members in the TCGA data set were figured out using the Pearson correlation analysis. Then, the top 50 co‐expression genes of S100A family members were subjected to pathway enrichment analysis in ConsensuspathDB.

### 2.7 CD8^+^ T cells infiltration in PC

With the help of GSVA, single‐sample gene set enrichment analysis (ssGSEA) was performed to quantify the activity or enrichment levels of CD8^+^ T cells (CD8+ T cells infiltration and Cytolytic activity) in the TCGA PC cohort.^22^, ^23^ Then, we investigated the differences in the infiltration and cytolytic activity of CD8^+^ T cell between the high‐ and low‐risk groups. Besides, correlation analysis between S100A family and the CD8^+^ T infiltration and cytolytic activity in PC was performed with Pearson correlation coefficients.

### 2.8 Statistical analysis

R software (http:///www.r-project.org/), SPSS 25.0 software and GraphPad prism 8.0 software (GraphPad Software, Inc) were used for statistical analyses. Correlations were calculated using Pearson correlated coefficient. Group differences were analysed by Wilcoxon test or Kruskal‐wallis test. *P* values <.05 were considered as statistically significant.

## 3 RESULTS

### 3.1 The mRNA expression of S100A family members in PC

**FIGURE 1:**
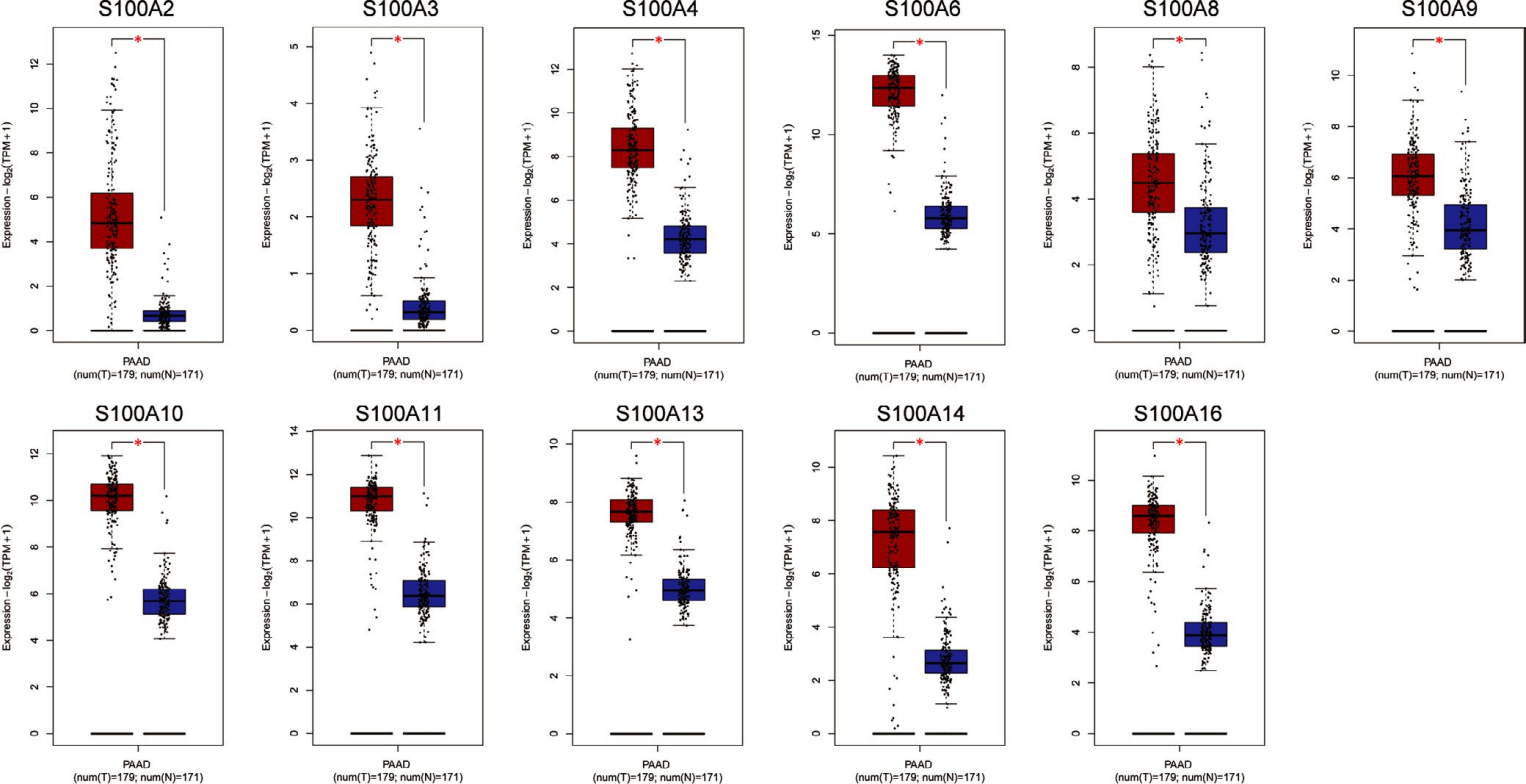
Differentially expressed S100A family members in GEPIA database. T, tumour; N, normal

**FIGURE 2:**
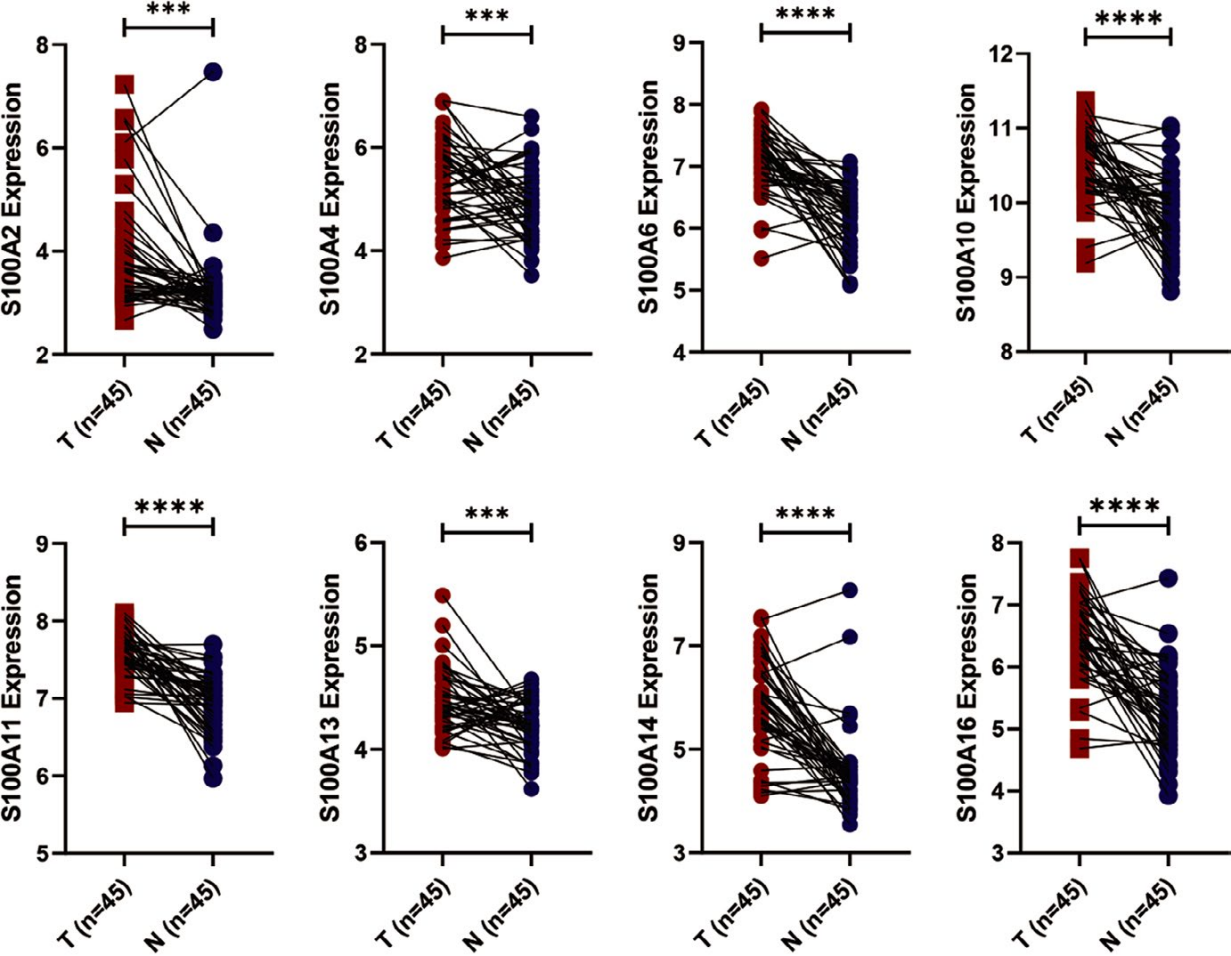
Paired differentially expressed S100A family members in GSE28735 dataset. T, tumour; N, normal. (*P value <.05; **P value <.01;***P value <.001; ****P value <.0001)

Based on the GEPIA database, we found that eleven among S100A family members were significantly up‐regulated in PC, including S100A2, S100A3, S100A4, S100A6, S100A8, S100A9, S100A10, S100A11, S100A13, S100A14 and S100A16 (Figure 1). Furthermore, based on GSE28735 data set, our study demonstrated that the expression of eight among the eleven differentially expressed S100A family members in PC tissues was up‐regulated compared with those in the adjacent non‐tumour tissues, including S100A2, S100A4, S100A6, S100A10, S100A11, S100A13, S100A14 and S100A16 (Figure 2). Taken together, the eight S100A family members were considered as potential oncogenes in PC and selected for further analysis.

### 3.2 KM survival analysis of S100A family members in PC

**FIGURE 3:**
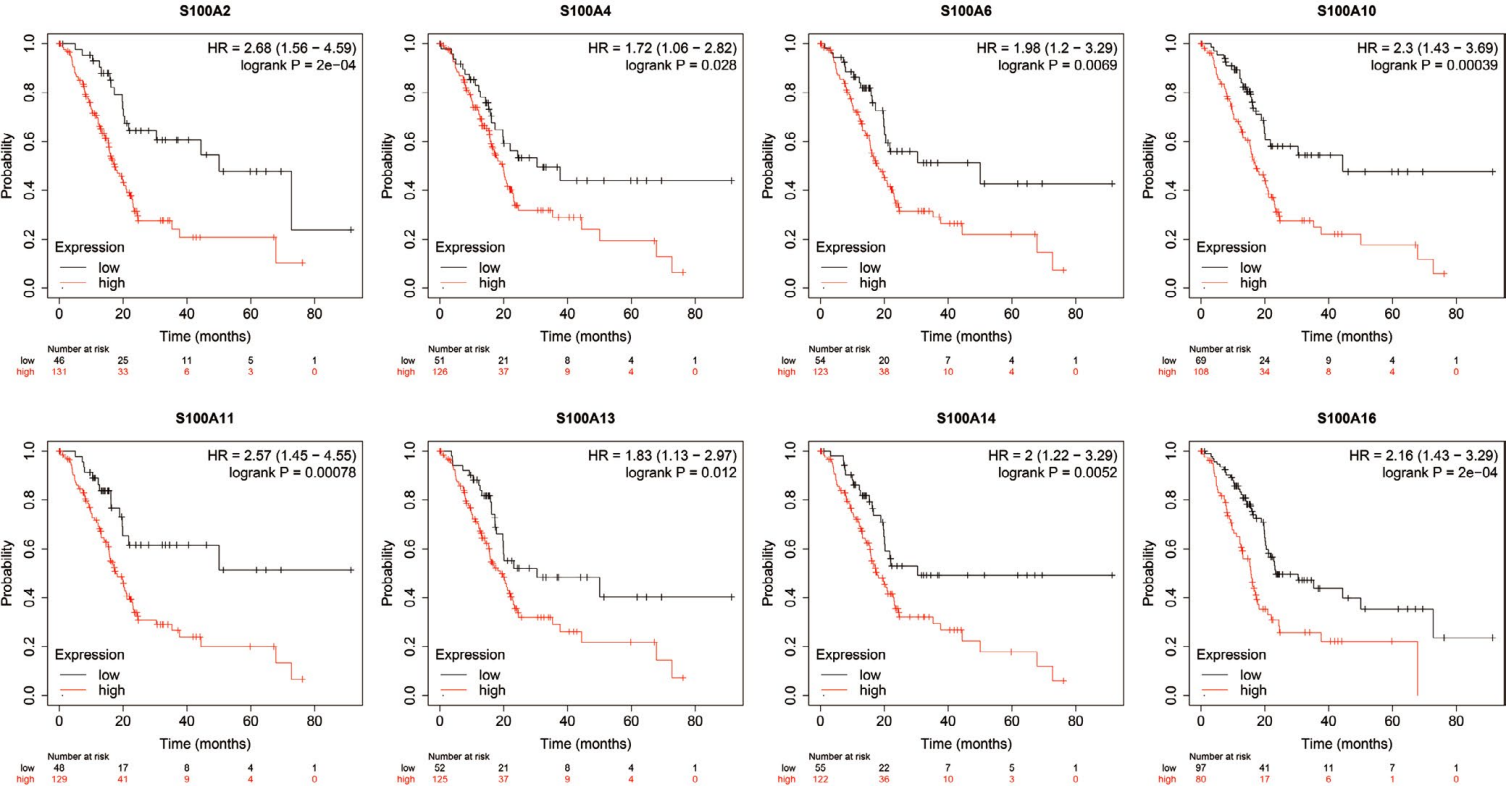
The survival curve for OS of the differentially expressed S100A family members in the KM plotter database. OS, overall survival; KM, Kaplan‐Meier

**FIGURE 4:**
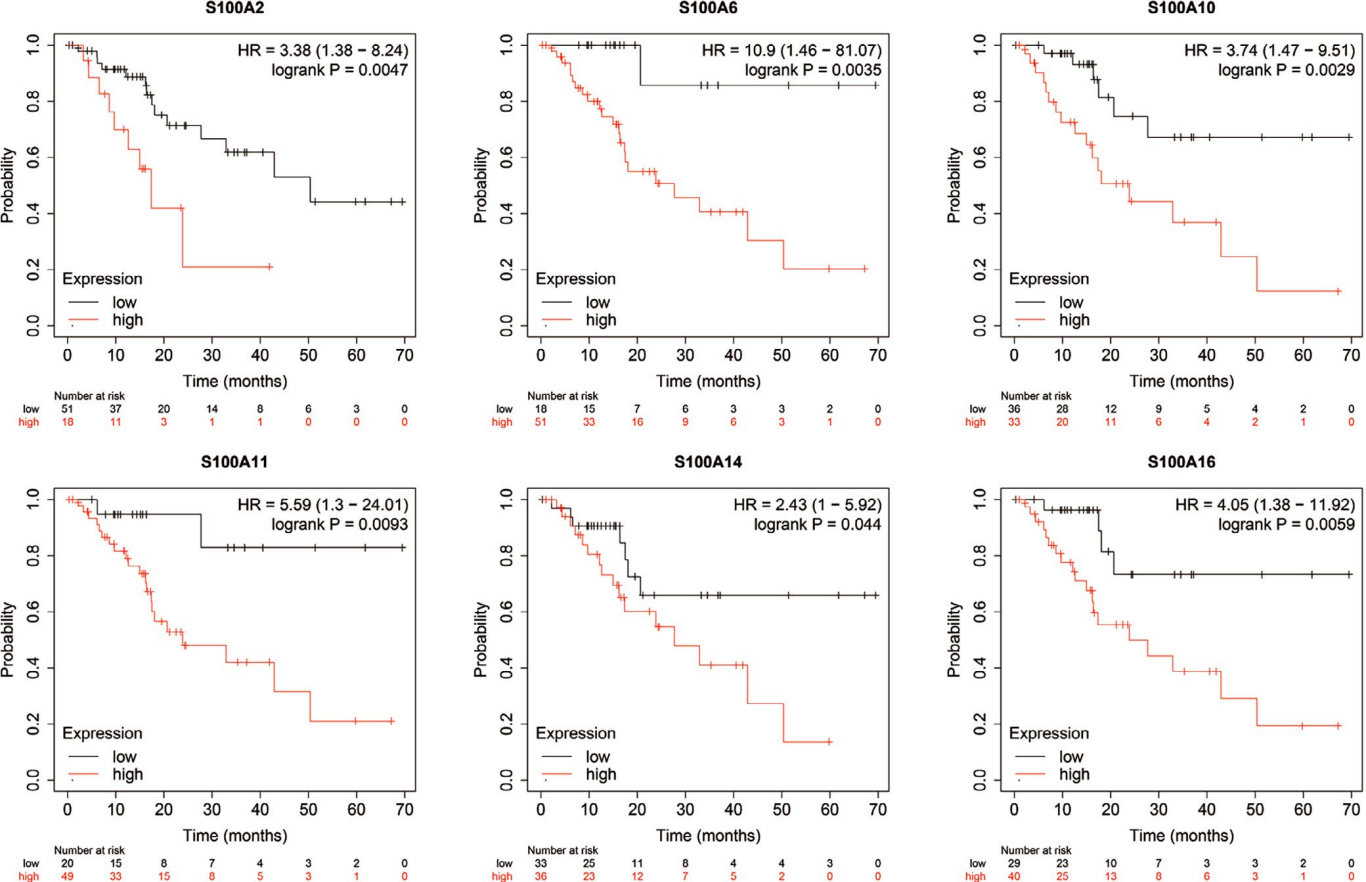
The survival curve for RFS of the differentially expressed S100A family members in the KM plotter database. RFS, recurrence‐free survival; KM, Kaplan‐Meier

To determine the survival effect of S100A family members (eg S100A2, S100A4, S100A6, S100A10, S100A11, S100A13, S100A14 and S100A16) in PC patients, we examined the correlation between mRNA expression and OS or RFS on log‐rank test using KM plotter database. KM curves for OS demonstrated that patients with lower expression of S100A2, S100A4, S100A6, S100A10, S100A11, S100A13, S100A14 and S100A16 had superior OS than those with higher expression of S100A2, S100A4, S100A6, S100A10, S100A11, S100A13, S100A14 and S100A16 (Figure 3). KM curves for RFS demonstrated that patients with higher expression of S100A2, S100A6, S100A10, S100A11, S100A14 and S100A16 had shorter RFS that those with lower expression of S100A2, S100A6, S100A10, S100A11, S100A14 and S100A16 (Figure 4). These results indicated that S100A2, S100A6, S100A10, S100A11, S100A14 and S100A16 were unfavourable prognostic factors in PC.

### 3.3 S100A family members in TNM stage and histologic grade

**FIGURE 5:**
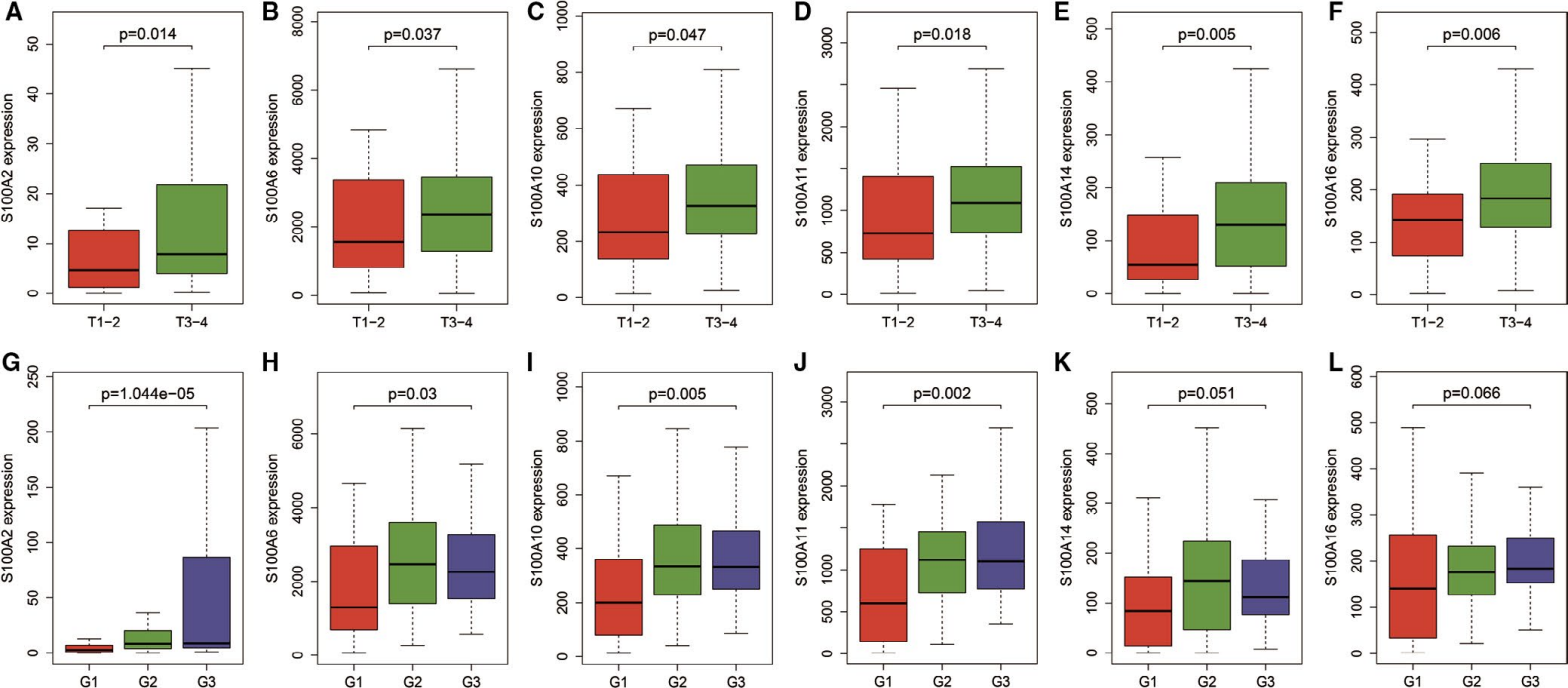
The mRNA expression of S100A2, S100A6, S100A10, S100A11, S100A14, and S100A16 in T stage and histologic grade. A‐F, The mRNA expression of S100A2, S100A6, S100A10, S100A11, S100A14, and S100A16 were significantly increased in patients with T3‐4 than in patients with T1‐2. G‐L, Higher mRNA expression of S100A2, S100A6, S100A10, S100A11, S100A14, and S100A16 were also observed in patients with higher histologic grade, though not statistically significant for S100A14 and S100A16. G1: grade 1; G2: grade 2; G3: grade3

The mRNA expression of S100A2, S100A6, S100A10, S100A11, S100A14 and S100A16 was significantly increased in patients with T3‐4 than in patients with T1‐2 (Figure 5A‐F), though not significantly associated with N stage and M stage. Furthermore, higher mRNA expression of S100A2, S100A6, S100A10, S100A11, S100A14 and S100A16 were also observed in patients with higher histologic grade, though not statistically significant for S100A14 and S100A16 (Figure 5G‐L). These results indicated that overexpression of S100A2, S100A6, S100A10, S100A11, S100A14 and S100A16 was related with tumour progression in PC.

### 3.4 Prognostic values of S100A2, S100A6, S100A10, S100A11, S100A14 and S100A16 DNA methylation

**FIGURE 6:**
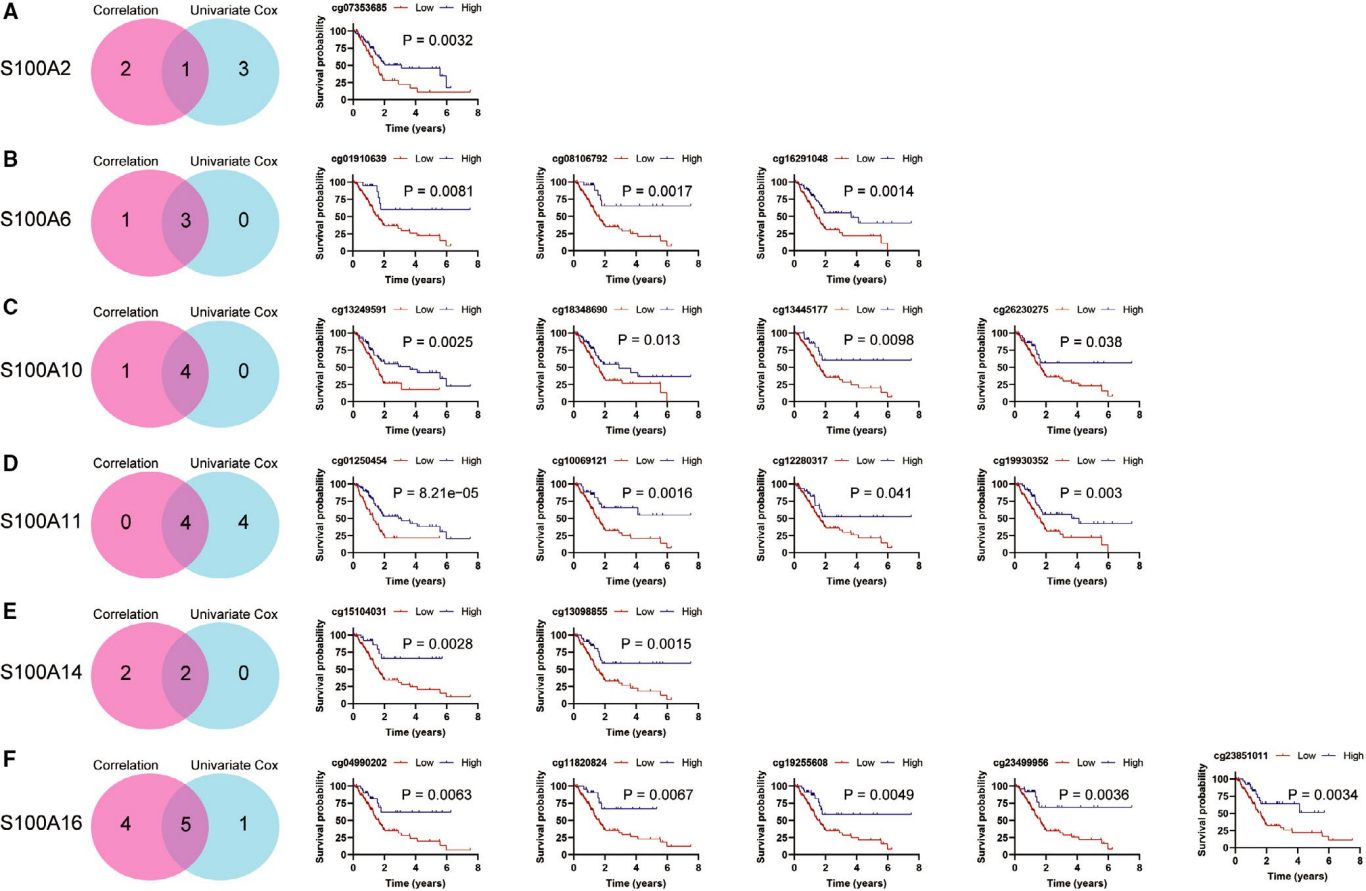
KM survival curves for OS of critical CpGs of S100A2, S100A6, S100A10, S100A11, S100A14, and S100A16. A, One critical CpG of S100A2 was positively associated with OS of PC. B, Three critical CpGs of S100A6 were positively associated with OS of PC. C, Four critical CpGs of S100A10 were positively associated with OS of PC. D, Four critical CpGs of S100A11 were positively associated with OS of PC. E, Two critical CpGs of S100A14 were positively associated with OS of PC. F, Five critical CpGs of S100A16 were positively associated with OS of PC. PC, pancreatic cancer; OS, overall survival; KM, Kaplan‐Meier

First, correlation analysis between the mRNA expression of S100A family members and the corresponding CpG methylation in PC demonstrated that three CpG of S100A2, four CpG of S100A6, five CpG of S100A10, four CpG of S100A11, two CpG of S100A14 and five CpG of S100A16 were negatively associated with the mRNA expression of corresponding S100A family members (Cor <−0.4, *P* value <.0001) (Table 1). Then, univariate Cox analysis demonstrated that four CpG of S100A2, three CpG of S100A6, four CpG of S100A10, eight CpG of S100A11, two CpG of S100A14 and six CpG of S100A16 were associated with significant prognosis (Table 2). Then, we figured out one critical CpG of S100A2, three critical CpG of S100A6, four critical CpG of S100A10, four critical CpG of S100A11, two critical CpG of S100A14 and five critical CpG of S100A16. KM survival analysis demonstrated that all of these critical CpG were positively associated with OS of patients with PC (Figure 6).

**TABLE 1:** Correlation between the CpG methylation level and the mRNA expression of S100A family member

Gene	CpG	Cor	*P* value
S100A2	cg00647881	−0.66	<.00001
S100A2	cg07353685	−0.52	<.00001
S100A2	cg27310485	−0.65	<.00001
S100A6	cg01910639	−0.58	<.00001
S100A6	cg08106792	−0.55	<.00001
S100A6	cg16291048	−0.62	<.00001
S100A6	cg24375627	−0.55	<.00001
S100A10	cg04989070	−0.40	<.00001
S100A10	cg13249591	−0.42	<.00001
S100A10	cg13445177	−0.59	<.00001
S100A10	cg18348690	−0.45	<.00001
S100A10	cg26230275	−0.45	<.00001
S100A11	cg01250454	−0.57	<.00001
S100A11	cg10069121	−0.56	<.00001
S100A11	cg12280317	−0.53	<.00001
S100A11	cg19930352	−0.64	<.00001
S100A14	cg13098855	−0.51	<.00001
S100A14	cg15104031	−0.41	<.00001
S100A16	cg04990202	−0.59	<.00001
S100A16	cg11820824	−0.53	<.00001
S100A16	cg19255608	−0.56	<.00001
S100A16	cg23499956	−0.48	<.00001
S100A16	cg23851011	−0.57	<.00001

**TABLE 2:** Prognosis‐associated CpGs of S100A family member based on univariate Cox analysis

Gene	CpG	HR	HR.95% L	HR.95% H	*P* value
S100A2	cg02332117	0.069	0.009	0.544	.011
S100A2	cg07353685	0.092	0.013	0.663	.018
S100A2	cg19907725	0.161	0.038	0.685	.013
S100A2	cg22700686	0.118	0.026	0.536	.006
S100A6	cg01910639	0.120	0.025	0.569	.008
S100A6	cg08106792	0.136	0.024	0.768	.024
S100A6	cg16291048	0.185	0.054	0.631	.007
S100A10	cg13249591	0.005	0.000	0.318	.013
S100A10	cg13445177	0.317	0.104	0.970	.044
S100A10	cg18348690	0.130	0.023	0.747	.022
S100A10	cg26230275	0.139	0.025	0.784	.025
S100A11	cg01250454	0.161	0.043	0.603	.007
S100A11	cg01603146	0.000	0.000	0.682	.040
S100A11	cg06767701	0.000	0.000	0.061	.022
S100A11	cg10069121	0.031	0.004	0.260	.001
S100A11	cg12280317	0.088	0.015	0.527	.008
S100A11	cg16382778	0.004	0.000	0.787	.041
S100A11	cg19930352	0.176	0.055	0.563	.003
S100A11	cg24067813	0.000	0.000	0.241	.021
S100A14	cg13098855	0.206	0.052	0.812	.024
S100A14	cg15104031	0.115	0.015	0.899	.039
S100A16	cg04990202	0.262	0.095	0.723	.010
S100A16	cg11820824	0.336	0.116	0.970	.044
S100A16	cg19255608	0.125	0.027	0.573	.007
S100A16	cg23499956	0.095	0.013	0.720	.023
S100A16	cg23851011	0.282	0.082	0.965	.044
S100A16	cg24843511	61.297	2.624	1431.882	.010

### 3.5 Prognostic signature based on critical CpG of S100A family members

**FIGURE 7:**
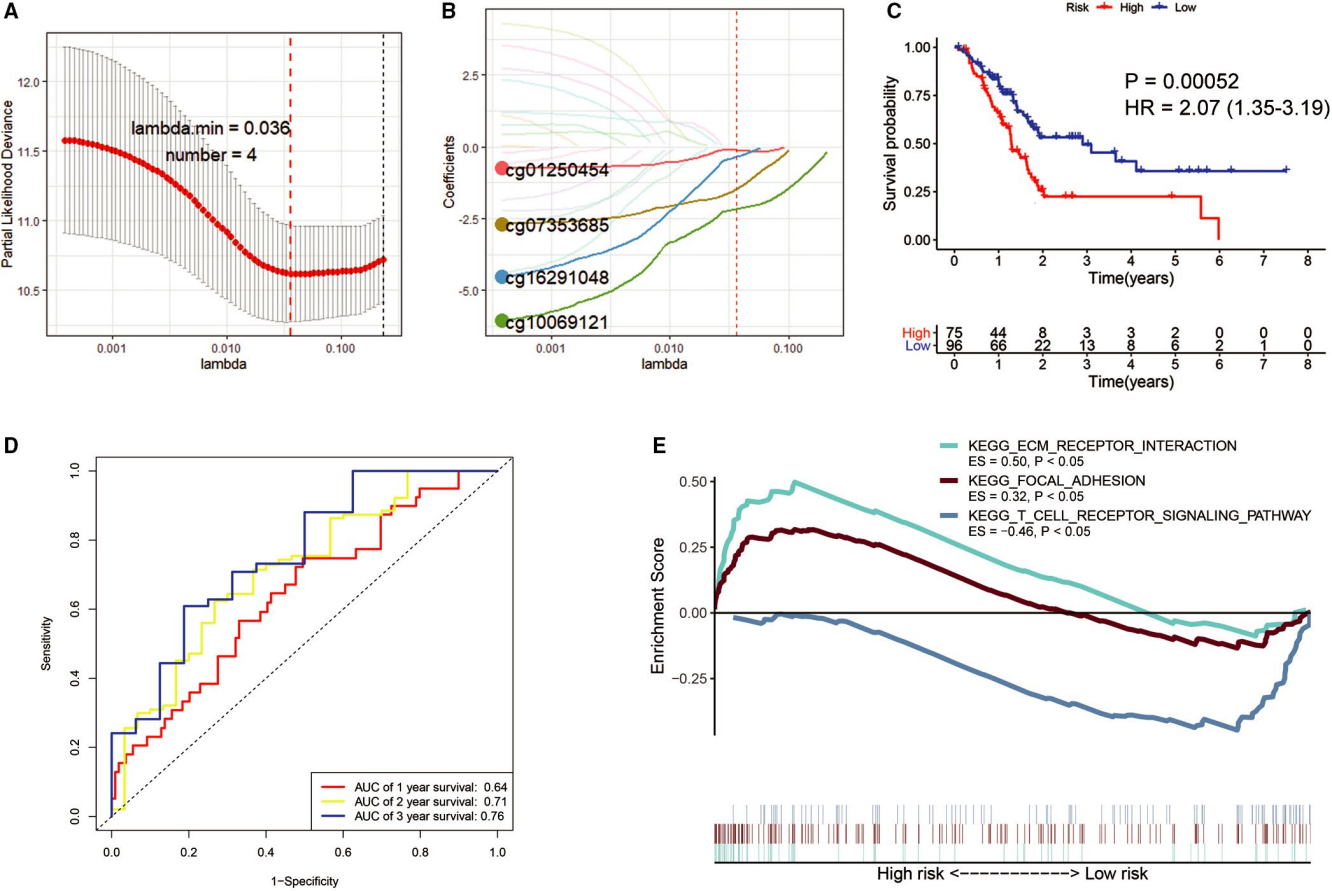
Establishment of A prognostic signature based on four critical CpGs. (A and B, Laoss regression analysis identified four critical CpGs to establish a prognostic signature for OS of PC. C, KM survival curves demonstrated that the low‐risk group displayed a significantly favourable OS than the high‐risk group. D, ROC curve analysis of the signature for 1‐, 2‐ and 3‐year OS prediction of PC patients. E, GSEA demonstrated that ECM‐receptor interaction and focal adhesion‐related gene sets were enriched in the high‐risk group, whereas T cell receptor signalling pathway‐related gene set were enriched in the low‐risk group

Using Lasso regression analysis, we identified four Critical CpG for establishing a prognostic signature (Figure 7A,B). For each individual in the TCGA PC data set, a prognostic risk score was calculated based on multivariate Cox regression analysis. Risk score = (−2.4399 × methylation of cg07353685) + (−0.8511 × methylation of cg16291048) + (−2.6501 × methylation of cg10069121) + (0.0099 × methylation of cg01250454). All cases were divided into a high‐risk group (score > 1.1817) and a low‐risk group (score < 1.1817) according to the optimal cut‐off value. The low‐risk group showed a significantly superior OS than the high‐risk group (HR = 2.07, 95% CI: 1.35‐3.19, *P* value = .00052) (Figure 7C). The C‐indexes for OS prediction with this prognostic signature were 0.61 (95% CI, 0.54‐0.68). Of note, the AUC value of the prognostic signature increased to 0.76 as follow‐up periods increased (Figure 7D). Furthermore, we found that ECM‐receptor interaction and focal adhesion‐related gene sets were significantly enriched in the high‐risk group, whereas T cell receptor signalling pathway‐related gene set was significantly enriched in the low‐risk group (Figure 7E).

### 3.6 Pathway enrichment analysis of S100A2, S100A6, S100A10, S100A11, S100A14 and S100A16

**FIGURE 8:**
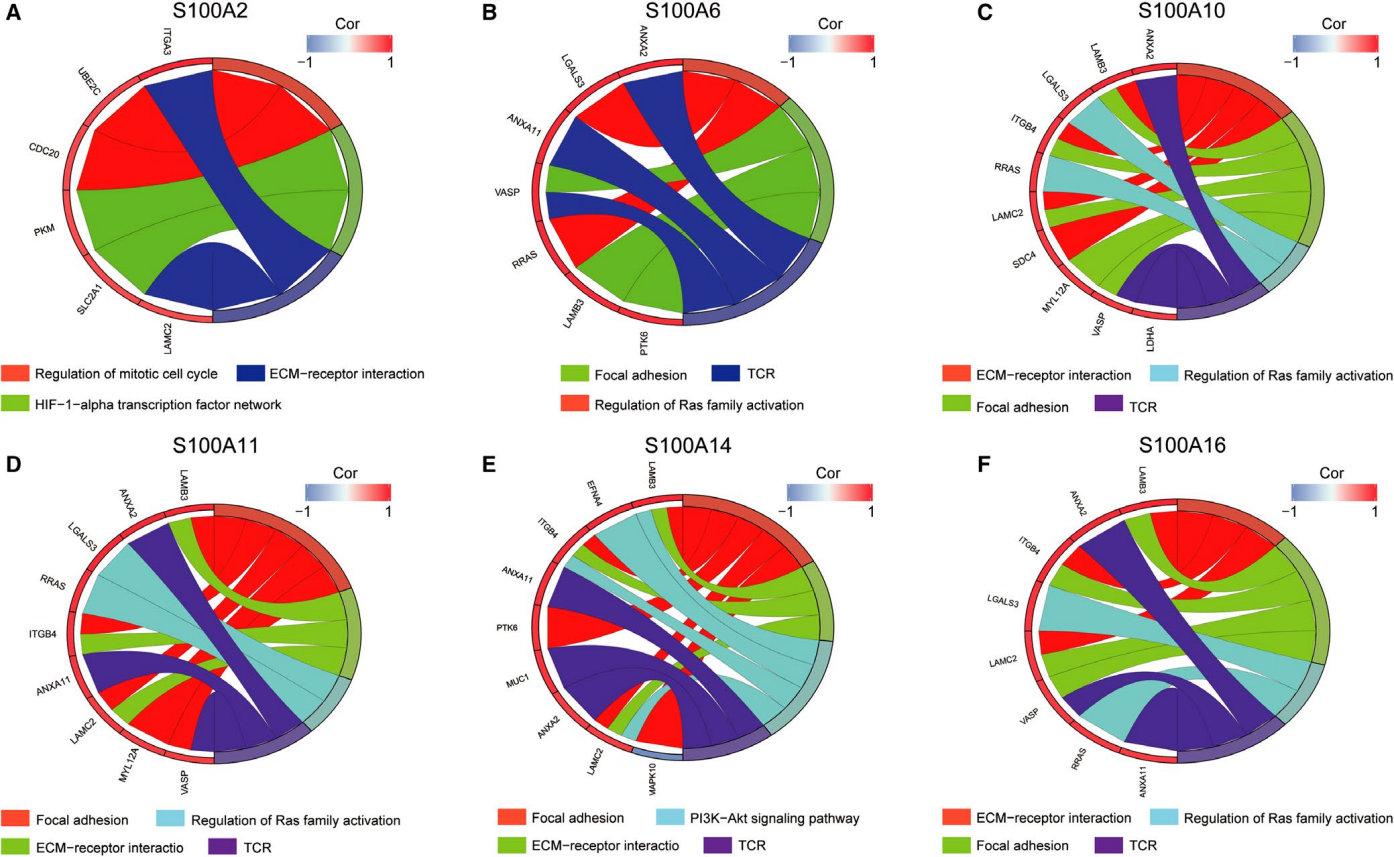
Pathway enrichment analysis of S100A2, S100A6, S100A10, S100A11, S100A14, and S100A16. ECM, extracellular matrix; TCR, T cell receptor signalling pathway

The top 50 co‐expressed genes of S100A2, S100A6, S100A10, S100A11, S100A14 and S100A16 were respectively enrolled into ConsensuspathDB and subjected to pathway enrichment analysis. S100A2 was significantly involved in the regulation of mitotic cell cycle, ECM‐receptor interaction and HIF‐1α transcription factor network (Figure 8A). Interestingly, S100A6, S100A10, S100A11, S100A14 and S100A16 may play an crucial role in focal adhesion, Ras‐stimulated signalling of PI3K‐Akt and T cell receptor signalling pathway (Figure 8B‐F). Thus, these results suggested that S100A family members (eg S100A6, S100A10, S100A11, S100A14 and S100A16) may significantly associated with the immune infiltration of TME in PC.

### 3.7 Higher risk score and overexpression S100A family members associated with less CD8^+^ T cell infiltration

**FIGURE 9:**
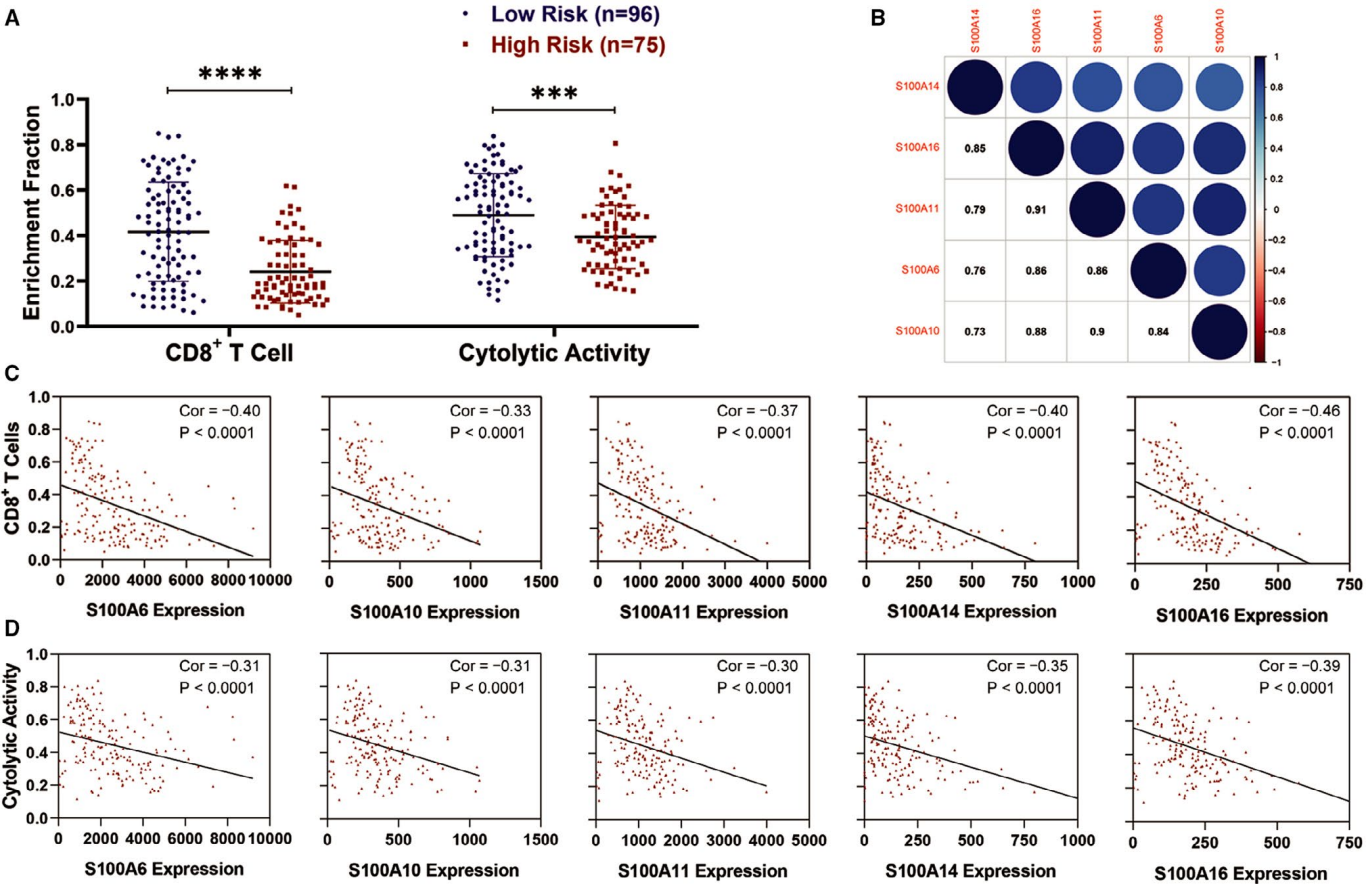
The infiltration and cytolytic activity of CD8+ T cell in the TME of PC. A, The enrichment fraction of CD8+ T cell infiltration and cytolytic activity in high‐risk group were significantly lower than those in low‐risk group. B, The mRNA expression correlation among S100A6, S100A10, S100A11, S100A14, and S100A16. C, The infiltration of CD8+ T cell was negatively associated with the expression of S100A6, S100A10, S100A11, S100A14, and S100A16. D, The cytolytic activity of CD8+ T cell was negatively associated with the expression of S100A6, S100A10, S100A11, S100A14, and S100A16. TME, tumour microenvironment; PC, pancreatic cancer

Considering that T cell receptor signalling pathway was negative associated with the CpG based signature, we further evaluated the differences in the infiltration and cytolytic activity of CD8^+^ T cell between high‐ and low‐risk groups. Using ssGSEA, we found that the enrichment fraction of CD8^+^ T cell infiltration and cytolytic activity in low‐risk group were significantly higher than those in high‐risk group (Figure 9A). Of note, we found that S100A6, S100A10, S100A11, S100A14 and S100A16 were highly co‐expressed with each other, indicating the similar potential biological functions among them (Figure 9B). ssGSEA also demonstrated that the infiltration and cytolytic activity of CD8^+^ T cell were negatively associated with the expression of S100A6, S100A10, S100A11, S100A14 and S100A16 (Figure 9C,D). Furthermore, using GEPIA database, we found that high expression of S100A family members (eg S100A6, S100A10, S100A11, S100A14 and S100A16) significantly associated with low expression of the marker genes from TILs (eg CD8A, CD8B, CD2, CD3D and CD3E) in PC (Table 3).

**TABLE 3:** Correlation between S100A family members and marker genes of TILs in GEPIA

Gene Markers	S100A6		S100A10		S100A11		S100A14		S100A16	
	Cor	*P* value	Cor	*P* value	Cor	*P* value	Cor	*P* value	Cor	*P* value
CD8A	−0.4	3.80E‐08	−0.29	7.50E‐05	−0.34	4.60E‐06	−0.36	6.70E‐07	−0.37	3.00E‐07
CD8B	−0.37	3.60E‐07	−0.26	0.00049	−0.31	2.10E‐05	−0.35	1.40E‐06	−0.31	1.80E‐05
CD2	−0.36	8.70E‐07	−0.26	0.00038	−0.31	2.40E‐05	−0.32	9.10E‐06	−0.35	1.40E‐06
CD3D	−0.21	5.40E‐03	−0.2	0.0077	−0.22	0.0027	−0.21	0.0053	−0.22	0.0026
CD3E	−0.3	4.40E‐05	−0.23	0.0023	−0.28	0.00018	−0.27	0.00024	−0.28	0.00011

## 4 DISCUSSION

In this study, we aimed to demonstrate the roles of S100A family members in tumour progression and immunosuppression regulation in PC. The S100A family was significantly overexpressed in PC and significantly associated with higher T stage, advanced histologic grade and poorer survival of PC patients, especially for S100A2, S100A6, S100A10, S100A11, S100A14 and S100A16. These results suggest that S100A family members may serve as potential prognostic biomarkers for PC.

Our study evaluated the whole picture of the significantly correlated and prognostic CpG of S100A family in PC, and we identified 19 critical CpG of S100A family in PC, including cg07353685 of S100A2, cg01910639 of S100A6, cg08106792 of S100A6, cg16291048 of S100A6, cg13249591 of S100A10, cg13445177 of S100A10, cg18348690 of S100A10, cg26230275 of S100A10, cg01250454 of S100A11, cg10069121 of S100A11, cg12280317 of S100A11, cg19930352 of S100A11, cg13098855 of S100A14, cg15104031 of S100A14, cg04990202 of S100A16, cg11820824 of S100A16, cg19255608 of S100A16, cg23499956 of S100A16 and cg23851011 of S100A16. Higher methylation of these 19 critical CpG were significantly correlated with lower expression of corresponding S100A family members and better OS of PC patients. Consistently, Bydoun et al reported that S100A10 expression is regulated through hypomethylation at specific CpG, such as cg13445177 and cg1324951.^16^ However, up to now, there is little methylation‐related study about S100A2, S100A6, S100A11, S10014 and S100A16 in PC. For the first time, our study suggest that the hypomethylation of these 19 critical CpG perhaps promotes the overexpression of corresponding S100A family members.

Furthermore, the prognostic signature based on the critical CpG cg07353685, cg16291048, cg10069121 and cg01250454 showed good prediction values for PC patients. Patients in lower‐risk group had a better OS compared with those in higher‐risk group. And ECM‐receptor interaction and focal adhesion‐related gene sets were highly enriched in the high‐risk group, whereas T cell receptor signalling pathway‐related gene set were highly enriched in the low‐risk group. We also found that the enrichment fraction of CD8^+^ T cell infiltration and cytolytic activity in low‐risk group were significantly higher than those in high‐risk group. Taken together, the critical CpG‐based signature facilitated clinicians to accurately predict the prognosis and evaluate the infiltration and anti‐tumour activity of CD8^+^ T cells in the TME of PC patients and choose more appropriate postoperative therapy individually.

Through pathway enrichment analysis, we figured out that S100A2 may take part in the regulation of mitotic cell cycle, ECM‐receptor interaction and HIF‐1α transcription factor network. Ohuchida et al demonstrated that S100A2 overexpression in PC associates with tumour progression and poor prognosis.^15^ And Biankin et al identified S100A2 as a useful predictor of response to radical surgery for PC and S100A2 overexpression as a metastatic phenotype marker.^24^ A previous study by Wen et al reported that S100A2 expression is significantly down‐regulated in the radioresistant PC cells, indicating S100A2 may be one of the molecular mechanisms of radioresistance of PC cells.^25^ However, little researches explored the potential function of S100A2 in pancreatic carcinogenesis. Our study is the first to demonstrate that S100A2 overexpression may promote tumour progression through regulating ECM‐receptor interaction, and HIF‐1α transcription factor network in PC, which provides important rational for future experimental study about S100A2.

The present study demonstrated that S100A6, S100A10, S100A11, S100A14 and S100A16 may play a vital role in focal adhesion and Ras‐stimulated signalling of PI3K‐Akt. As the best of our knowledge, little is known about the potential role of S100A6, S100A10, S100A11, S100A14 and S100A16 in the formation of focal adhesion in PC. Of note, S100A4 overexpression was reported to promote PC progression through FAK‐mediated signalling pathway.^26^ Activation of FAK is positively correlated with the AJCC stage and histologic grade of PC. Besides, the formation and turnover of focal adhesion are critical for cell migration. Previous studies revealed that focal adhesion interacts with the ECM and can induce epithelial to mesenchymal transition (EMT), thereby promoting pancreatic carcinogenesis. Furthermore, activation of FAK is associated with the aggressive ability in PC through Ras‐stimulated signalling of PI3K‐Akt, which is active in PC.^27^ Xiao et al revealed that S100A11 is involved in the PI3K‐Akt signalling pathway.^19^ Taken together, we proposed that S100A6, S100A10, S100A11, S100A14 and S100A16 promote PC progression through focal adhesion‐Ras‐stimulated signalling pathway.

Pathway enrichment analysis also revealed that S100A6, S100A10, S100A11, S100A14 and S100A16 may be associated with T cell receptor signalling pathway, indicating their association with TIL infiltration in the TME of PC. In addition, ssGSEA revealed that S100A6, S100A10, S100A11, S100A14 and S100A16 overexpression significantly correlated with low infiltration and cytolytic activity of CD8^+^ T cells. Co‐expression analysis also demonstrated that S100A6, S100A10, S100A11, S100A14 and S100A16 overexpression were significantly associated with lower expression of the marker genes from TILs (eg CD8A, CD8B, CD2, CD3D and CD3E). These results further suggested the potential functions of S100A6, S100A10, S100A11, S100A14 and S100A16 in decreasing the infiltration and anti‐tumour activity of CD8^+^ T cells.

Focal adhesion signalling pathway is widely considered as one of the oncologic cell‐intrinsic pathway influencing tumour immunity.^28^ Jiang et al reported that FAK1 overexpression in PC significantly associates with poorer infiltration of CD8^+^ T cells.^29^ Targeting FAK could render PC responsive to ICI.^29^ Combination of FAK inhibitor with PD‐1 blockade and gemcitabine is currently being investigated in clinical trials for PC.^29^ In addition, formation and turnover of focal adhesion can also promote EMT, which induces immunosuppression in the TME.^30^, ^31^, ^32^ Increased EMT was also reported to be associated with resistance to ICI in solid tumours.^33^, ^34^ Activation of Ras‐stimulating signalling pathway also induces the immunosuppressive TME of PC.^35^ KRAS‐mutated PC cells could induce the expression of granulocyte‐macrophage colony‐stimulating factor (GM‐CSF) and recruit neutrophils and myeloid cells, both of which inhibit the infiltration and cytolytic activities of CD8^+^ T cells.^36^, ^37^, ^38^ Taken together, S100A6, S100A10, S100A11, S100A14 and S100A16 overexpression may suppress CD8+ T cells infiltration and cytolytic activities through focal adhesion‐Ras‐stimulated signalling pathway. Targeting S100A6, S100A10, S100A11, S100A14, and S100A16 may block focal adhesion‐Ras‐stimulating signalling, which could be a promising therapeutic strategy complementary to current immunotherapies and efficiently improve their effectiveness.

To the best of our knowledge, we are the first to systematically describe the prognostic values of the mRNA expression and the DNA methylation of S100A family in PC. Moreover, for the first time, we demonstrated that S100A6, S100A10, S100A11, S100A14 and S100A16 may suppress CD8^+^ T cells infiltration and cytolytic activities through focal adhesion‐Ras‐stimulated signalling pathway. However, some limitations should be acknowledged in this study. The first limitation of this study is the lack of experimental validation and externally clinical cohort validation. Second, the study outcome was largely dependent on the quality of data from publicly available databases.

## 5 CONCLUSIONS

In the current study, we comprehensively described the prognostic value and potential biological functions of S100A family members in PC, providing insights for further investigation of S100A family members as potential targets in PC.

## CONFLICT OF INTEREST

The authors declare that they have no potential conflict of interest.

## AUTHOR CONTRIBUTIONS

**Hongkai Zhuang:** Conceptualization (lead); Data curation (lead); Formal analysis (lead); Investigation (lead); Methodology (lead); Project administration (lead); Software (lead); Supervision (lead); Validation (lead); Visualization (lead); Writing‐original draft (lead); Writing‐review & editing (lead). **Xinming Chen:** Formal analysis (equal); Methodology (equal); Validation (equal); Visualization (equal); Writing‐original draft (equal). **Fengying Dong:** Software (equal); Writing‐review & editing (equal). **Zedan Zhang:** Methodology (supporting); Writing‐original draft (supporting). **Zixuan Zhou:** Methodology (supporting). Zuyi Ma: Methodology (supporting). **Shanzhou Huang:** Data curation (supporting); Methodology (supporting). **Bo Chen:** Conceptualization (equal). **Chuanzhao Zhang:** Conceptualization (equal); Funding acquisition (equal); Writing‐original draft (equal); Writing‐review & editing (equal). **Baohua Hou:** Conceptualization (equal); Funding acquisition (equal); Writing‐original draft (equal); Writing‐review & editing (equal).

## Data Availability

The authors confirm that the data supporting the findings of this study are available within the article and its supplementary.
